# Diffuse Alveolar Hemorrhage Associated With Severe Acute Respiratory Syndrome Coronavirus 2

**DOI:** 10.7759/cureus.20171

**Published:** 2021-12-05

**Authors:** Haytham A Wali, Deanne Tabb, Saeed A Baloch

**Affiliations:** 1 Department of Pharmacy Practice, College of Clinical Pharmacy, King Faisal University, Al-Ahsa, SAU; 2 Department of Pharmacy Services, Piedmont Columbus Regional Midtown, Columbus, USA; 3 Division of Infectious Diseases, Department of Medicine, Piedmont Columbus Regional Midtown, Columbus, USA

**Keywords:** autoimmune diseases, coronavirus disease-19, glomerulonephritis, anca-associated vasculitis, diffuse alveolar hemorrhage

## Abstract

Diffuse alveolar hemorrhage (DAH) is a rare syndrome resulting from the accumulation of intra-alveolar red blood cells originating most often from the alveolar capillaries and, less frequently, from precapillary arterioles or postcapillary venules. The causes of DAH can be divided into infectious and noninfectious. Severe acute respiratory syndrome coronavirus 2 (SARS-CoV-2) is a novel coronavirus that has not been previously identified in humans, and it is responsible for coronavirus disease-19 (COVID-19) infection. Here, we present a case of DAH that is believed to be a consequence of COVID-19 infection in a female patient with no known past medical history. The patient was found to be positive for perinuclear anti-neutrophil cytoplasmic antibodies (P-ANCA) and anti-glomerular basement membrane antibodies. The patient was diagnosed with ANCA-associated vasculitis with glomerulonephritis and was treated successfully with methylprednisolone 500 mg intravenous (IV) daily for three days, followed by rituximab 375 mg/m^2^ IV once weekly for four weeks. The long-term complications of COVID-19 are not entirely known and are still being investigated. The association between COVID-19 infection and DAH is not fully known. However, the inflammatory process of COVID-19 infection may have a role in vasculitis, leading to DAH.

## Introduction

Diffuse alveolar hemorrhage (DAH), commonly recognized by the signs of anemia, hemoptysis, diffuse radiographic pulmonary infiltrates, and hypoxemic respiratory failure, is a rare syndrome resulting from the accumulation of intra-alveolar red blood cells originating most often from the alveolar capillaries and, less frequently, from precapillary arterioles or postcapillary venules [1-3]. The prognosis of DAH is poor, with hospital mortality ranging from 20% to over 50% [4]. The causes of DAH can be divided into infectious and noninfectious. Pulmonary infections include those caused by viruses, bacteria, fungi, and parasites [3]. In immunocompromised patients, DAH has been reported due to cytomegalovirus [5], adenovirus [6], invasive aspergillosis [7], *Mycoplasma hominis* [8], *Legionella pneumophila* [9], and *Strongyloides stercoralis* [10]. Conversely, in immunocompetent patients, DAH was reported in infections due to influenza A (H1N1) virus [11], dengue fever [12], leptospirosis [13], and *Plasmodium falciparum* [14].

Severe acute respiratory syndrome coronavirus 2 (SARS-CoV-2) is a novel coronavirus that has not been previously identified in humans and is responsible for the coronavirus disease-19 (COVID-19) infection. The first case was identified in Wuhan, China, in December 2019, and it has spread to many countries around the world and was declared a global pandemic on March 11, 2020, by the World Health Organization (WHO) [15,16]. Cases of DAH after the COVID-19 infection have been reported in immunocompromised patients [17]. Here, we present a case of DAH that was believed to be a consequence of COVID-19 infection in a healthy, immunocompetent patient.

This case report was published previously as a preprint on July 26, 2021, through the Research Square preprinting server with the following digital object identifier (DOI): 10.21203/rs.3.rs-555436/v1.

## Case presentation

This report describes a 26-year-old Caucasian female with no known past medical history who presented to the Piedmont Columbus Regional Midtown emergency department with a chief complaint of worsening shortness of breath and dyspnea on exertion. The patient was found to be positive for COVID-19 around 25 days before this admission. The patient reported that she had isolated herself for 10 days during that time and that her symptoms had improved. However, one week before the current admission, she started experiencing worsening shortness of breath and cough with hemoptysis. These symptoms prompted her visit to an emergency department, where she was subsequently diagnosed with atypical pneumonia and discharged on doxycycline 100 mg orally every 12 hours for five days. 

The patient continued to worsen and was subsequently found to have generalized malaise, severe fatigue, nonproductive cough, myalgias, fever, decreased appetite, and hemoptysis in the current admission. The patient had no family history of bleeding disorders. Her vital signs on admission were the following: temperature, 100.8 °F (38.2 °C); blood pressure, 128/72 mmHg; heart rate, 122 beats per minute; respiratory rate, 35 breaths per minute; and oxygen (O_2_) saturation, 90% on room air, for which she required a 100% nonrebreather for oxygen support. An arterial blood gas panel was performed and revealed the following: pH of 7.43; pCO_2_ of 32.9 mmHg; pO_2_ of 73.6 mmHg; HCO_3_ of 21.9 mmol/L. The complete blood count on admission is presented in Table 1. The patient had a slightly elevated white blood cell count and severe anemia. Urinalysis was positive for protein and blood. All other laboratory values were unremarkable. The repeat COVID-19 test during this admission using the Xpert® Xpress SARS-CoV-2/Flu/RSV test (Cepheid®, Sunnyvale, CA, USA) and the SARS-COV-2 immunoglobulin G (IgG) antibody were positive, and the cycle threshold value was >33 cycles, which indicated that she was in the later stage of the infection.

**Table 1:** Complete blood count on admission. WBC = White blood cell.

Laboratory Test	Value	Normal Range
WBC, cells/mcL	11,200	4000–10,500
Hemoglobin, g/dL	6.9	12.0–16
Hematocrit, %	20.6	36.0–48.0
Platelet count, 10^3^/mcL	432	130–400

The chest computed tomography (CT) scan was negative for pulmonary embolism. However, it revealed extensive bilateral ground-glass opacities with confluent densities suggestive of multifocal pneumonia. Consequently, pulmonology and infectious diseases were consulted for evaluation and management. The patient was started on community-acquired pneumonia treatment with ceftriaxone 1 g intravenously (IV) every 24 hours for five days and azithromycin 500 mg IV every 24 hours for three days. Additionally, there was a strong suspicion for DAH, most likely due to a COVID-19-associated vasculitis process, for which methylprednisolone 250 mg IV four times per day was started. 

A series of serologic tests were ordered to investigate the cause of DAH. The findings are presented in Table 2. The patient was found to be positive for perinuclear anti-neutrophil cytoplasmic antibodies (P-ANCA). She was also found to be negative for antinuclear antibody (ANA), anti-double-stranded deoxyribonucleic acid, and cyclic citrullinated peptide antibodies. Based on the serologic findings, the patient was determined to have microscopic polyangiitis (MPA).

**Table 2:** The diffuse alveolar hemorrhage serologic findings. P-ANCA = perinuclear anti-neutrophil cytoplasmic antibodies; ANA = antinuclear antibody; IFA = immunofluorescence assay; CH50 = total complement.

Laboratory Test	Value	Normal Range
P-ANCA, Titer	1:40	<1:20
ANA Screen IFA	Negative	Negative
CH50, U/mL	49	31–60
C3 Complement, mg/dL	141	90–180
C4 Complement, mg/dL	16	10–40

At discharge, the patient had an oxygen saturation of 96% on room air and a respiratory rate of 18 breaths per minute with no evidence of ongoing hypoxia. The patient was prescribed a tapering course of prednisone (40 mg orally twice daily for five days, then 30 mg orally twice daily for five days, then 20 mg orally twice daily for five days, then 10 mg orally twice daily for five days, then 10 mg orally once daily for five days) and advised to follow up with both pulmonology and her primary care physician.

Three days after discharge, the anti-glomerular basement membrane (anti-GBM) antibodies titer, which was ordered during the hospital admission but sent out to an outside lab, was found to be positive (4.5 AI; normal range, <1.0 AI). In addition, the urinalysis obtained during admission confirmed the presence of blood and protein. The patient was informed about the positive results and advised to schedule an appointment with a nephrologist for further assessment.

**Figure 1:**
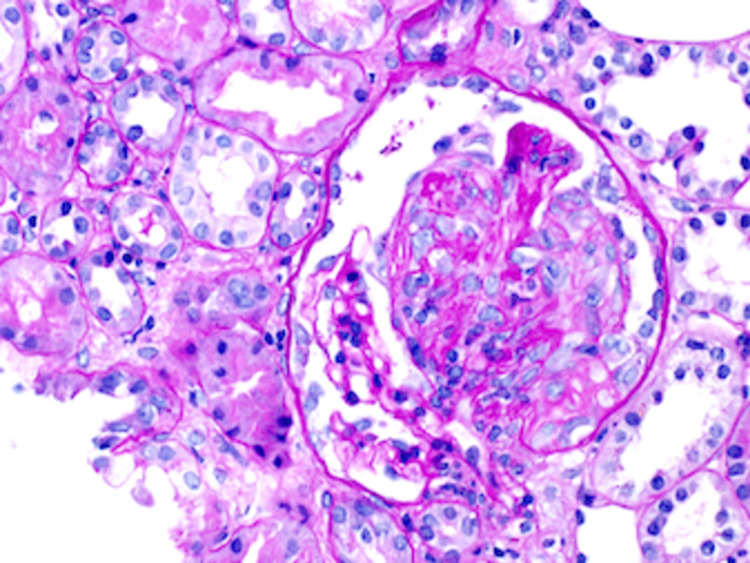
Light microscopy of the renal biopsy showing fibrocellular crescent.

**Figure 2:**
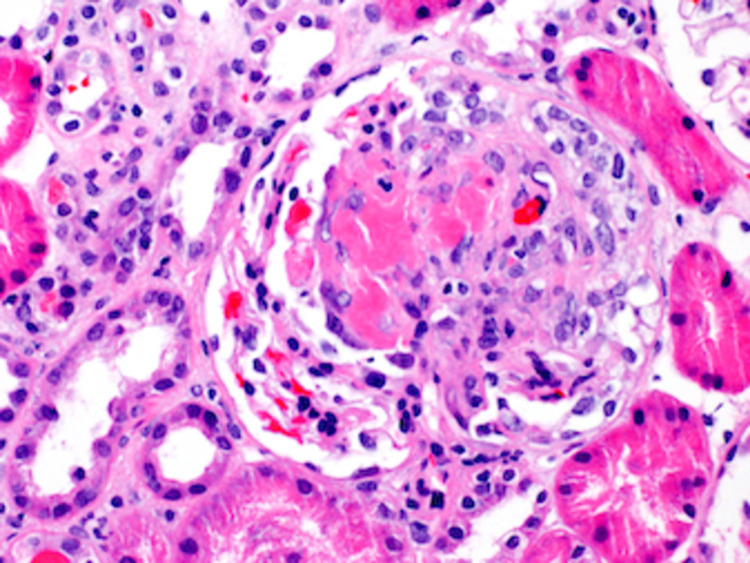
Light microscopy of the renal biopsy showing fibrinoid necrosis.

A renal biopsy was obtained during her visit with the nephrologist. The renal parenchyma available for light microscopic examination was approximately 90% cortex and 10% medulla. Forty-eight glomeruli were present, none of which were globally sclerotic. Two glomeruli showed segmental rupture of the capillary loops associated with fibrinoid necrosis of the glomerular tuft and cellular crescent formation. Twenty-six additional glomeruli showed areas of segmental glomerulosclerosis, 11 of which were associated with fibro-cellular crescents and 15 with fibrous crescents. The 20 uninvolved glomeruli showed no mesangial or endocapillary hypercellularity. The findings of the light microscopy can be seen in Figures 1, 2. 

**Figure 3:**
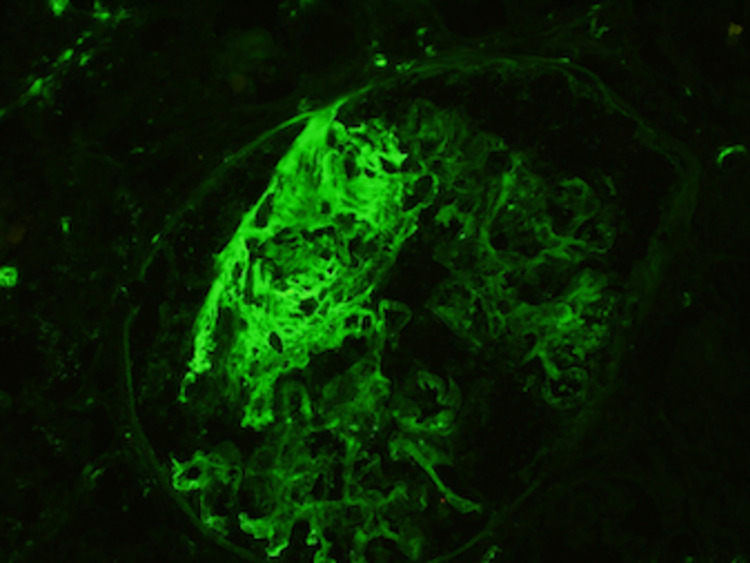
The immunofluorescence stain of the fibrinogen in the renal biopsy.

**Figure 4:**
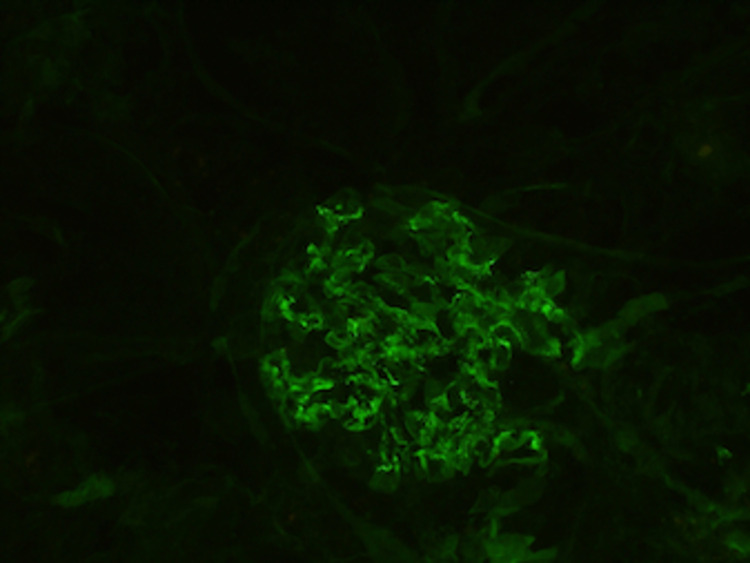
The immunofluorescence stain of the immunoglobulin G in the renal biopsy.

The renal parenchyma available for immunofluorescence studies was entirely represented by cortex and contained nine glomeruli, none of which were globally sclerotic. The sections were stained for IgG, IgM, IgA, C3, complement 1q, albumin, fibrinogen, kappa, and lambda light chains. In addition, there was focal intense fibrinogen staining within the glomerular tuft. Additionally, there was mild mesangial finely granular staining for IgA (trace), IgG (2+), kappa (2+), and lambda (1+). All other stains were negative within the glomeruli. The findings of the immunofluorescence studies can be seen in Figures 3, 4.

Based on the serology results mentioned above and the renal biopsy findings, the patient was determined to be having ANCA-associated vasculitis with glomerulonephritis and was started on methylprednisolone 500 mg IV daily for three days, followed by rituximab 375 mg/m^2^ IV once weekly for four weeks.

## Discussion

A variety of diseases and conditions can be associated with the development of DAH. One of the histopathologic patterns of DAH is pulmonary, or alveolar, capillaritis, which is characterized by the neutrophilic infiltration of the alveolar septa or the lung interstitium [4,18]. Although bronchoscopy with bronchoalveolar lavage is indicated in patients suspected of having DAH to confirm the diagnosis [19], this procedure was not performed in our case due to the risk of aerosolization and spread of COVID-19 [20]. 

ANCA-associated vasculitis is a group of autoimmune diseases including granulomatosis with polyangiitis (GPA) (formerly Wegener’s granulomatosis), eosinophilic GPA (formerly Churg-Strauss syndrome), and MPA. These diseases are characterized by inflammatory cell infiltration causing necrosis of small blood vessels [21]. Our patient was thought to have DAH caused by ANCA-associated vasculitis, but with the specific disease not wholly identified.

A positive ANCA test can indicate if DAH is due to GPA or MPA. Cytoplasmic ANCA, as determined by anti-proteinase 3 antibodies, is most consistent with GPA. At the same time, P-ANCA with positive anti-myeloperoxidase (anti-MPO) favors the diagnosis of MPA or GPA syndrome [22]. Anti-GBM antibodies are more specific to an anti-GBM disease or Goodpasture's syndrome, an autoimmune disorder characterized by autoantibodies directed against the GBM. However, 60% to 80% of the patients with anti-GBM disease have clinical manifestations of pulmonary and renal disease [23,24]. In addition, patients with acute glomerulonephritis with or without pulmonary hemorrhage also may have GPA or MPA. Thus, the serum should be tested for ANCA as well as anti-GBM antibodies. Anti-GBM disease and systemic vasculitis not only have similar clinical manifestations, but between 10% and 50% of patients with the anti-GBM disease also test positively for ANCA (usually anti-MPO-ANCA) at the time of diagnosis and may have signs of a systemic vasculitis or a marked systemic inflammatory response. Based on our patient's serologic findings throughout her admission, she was diagnosed initially with MPA. However, discovering the positive anti-GBM antibodies raised the suspicion for possible kidney involvement, which was subsequently confirmed by renal biopsy, even though the patient did not have any signs of kidney injury during her admission. 

COVID-19 infection is classified into three stages. It starts with early infection, then pulmonary involvement with or without hypoxia, and progresses to severe systemic hyper-inflammation. The late stage of COVID-19 is characterized by the elevation of systemic inflammation markers [24]. It has been reported that inflammatory cytokines and biomarkers such as interleukins (e.g., IL-2, IL-6, and IL-7), granulocyte colony-stimulating factor, macrophage inflammatory protein 1-a, tumor necrosis factor (TNF)-a, C-reactive protein, ferritin, and D-dimer are significantly elevated during this stage [25]. In addition, some of these inflammatory markers, including IL-6, IL-8, and TNF-a, are elevated as well with ANCA-associated vasculitis [26]. Like some autoimmune and immune-mediated thrombo-inflammatory diseases, neutrophil activation and neutrophil extracellular trap production (NETosis) appear to have a pathogenic role in COVID-19. SARS-CoV-2 infection can trigger cross-reactivity through molecular mimicry, leading to autoimmunity in patients with COVID-19 [27]. Since our patient was in the late stage of her COVID-19 infection, which was supported by the time since the onset of her COVID-19 symptoms, her positive SARS-COV-2 IgG antibodies, and her inflammatory markers, it was possible that the elevation of inflammatory markers at the time of admission could have contributed to the incidence of ANCA-associated vasculitis and, subsequently, the DAH and the kidney injury.

## Conclusions

We presented a rare case of DAH in a patient following infection with SARS-COV-2. The underlying cause is thought to be ANCA-associated vasculitis with glomerulonephritis. The association between COVID-19 infection and DAH is not fully known. However, the inflammatory process of COVID-19 infection may have a role in vasculitis, leading to DAH. Nevertheless, this case cannot establish a causal relationship between the two conditions, but it describes a clinical observation that might need to be considered when evaluating COVID-19 patients.

## References

[1] Leatherman JW, Davies SF, Hoidal JR (1984). Alveolar hemorrhage syndromes: diffuse microvascular lung hemorrhage in immune and idiopathic disorders. Medicine.

[2] de Prost N, Parrot A, Cuquemelle E (2012). Diffuse alveolar hemorrhage in immunocompetent patients: etiologies and prognosis revisited. Respir Med.

[3] von Ranke FM, Zanetti G, Hochhegger B, Marchiori E (2013). Infectious diseases causing diffuse alveolar hemorrhage in immunocompetent patients: a state-of-the-art review. Lung.

[4] Lara AR, Schwarz MI (2010). Diffuse alveolar hemorrhage. Chest.

[5] Ciledag A, Karnak D, Kayacan O (2010). A butterfly-shaped alveolar hemorrhage caused by cytomegalovirus. Southeast Asian J Trop Med Public Health.

[6] Mayeur N, Srairi M, Tetu L, Guilbeau Frugier C, Fourcade O, Dahan M (2012). Lethal hemorrhagic alveolitis after adenovirus pneumonia in a lung transplant recipient. Heart Lung.

[7] Agustí C, Ramirez J, Picado C (1995). Diffuse alveolar hemorrhage in allogeneic bone marrow transplantation. A postmortem study. Am J Respir Crit Care Med.

[8] Kane JR, Shenep JL, Krance RA, Hurwitz CA (1994). Diffuse alveolar hemorrhage associated with Mycoplasma hominis respiratory tract infection in a bone marrow transplant recipient. Chest.

[9] Kashif M, Patel R, Bajantri B, Diaz-Fuentes G (2017). Legionella pneumonia associated with severe acute respiratory distress syndrome and diffuse alveolar hemorrhage - a rare association. Respir Med Case Rep.

[10] Agarwal VK, Khurana HS, Le HX, Mathisen G, Kamangar N (2009). 30-year-old HIV-positive female with diffuse alveolar hemorrhage. J Intensive Care Med.

[11] Gilbert CR, Vipul K, Baram M (2010). Novel H1N1 influenza A viral infection complicated by alveolar hemorrhage. Respir Care.

[12] Marchiori E, Ferreira JL, Bittencourt CN (2009). Pulmonary hemorrhage syndrome associated with dengue fever, high-resolution computed tomography findings: a case report. Orphanet J Rare Dis.

[13] Luks AM, Lakshminarayanan S, Hirschmann JV (2003). Leptospirosis presenting as diffuse alveolar hemorrhage: case report and literature review. Chest.

[14] Saigal S, Kapoor G, Gurjar M, Singh DK (2014). Diffuse alveolar hemorrhage due to Plasmodium falciparum: a rare entity--are steroids indicated?. J Vector Borne Dis.

[15] Guan WJ, Ni ZY, Hu Y (2020). Clinical characteristics of coronavirus disease 2019 in China. N Engl J Med.

[16] (2021). World Health Organization. Coronavirus disease 2019 (COVID-19) Situation Report - 51. https://www.who.int/docs/default-source/coronaviruse/situation-reports/20200311-sitrep-51-covid-19.pdf?sfvrsn=1ba62e57_10.

[17] Löffler C, Mahrhold J, Fogarassy P, Beyer M, Hellmich B (2020). Two immunocompromised patients with diffuse alveolar hemorrhage as a complication of severe coronavirus disease 2019. Chest.

[18] Park MS (2013). Diffuse alveolar hemorrhage. Tuberc Respir Dis (Seoul).

[19] Ioachimescu OC, Stoller JK (2008). Diffuse alveolar hemorrhage: diagnosing it and finding the cause. Cleve Clin J Med.

[20] Krall J, Ali M, Maslonka M, Pickens A, Bellinger C (2020). Bronchoscopy in the COVID-19 era. Clin Pulm Med.

[21] Yates M, Watts R (2017). ANCA-associated vasculitis. Clin Med (Lond).

[22] Greco A, Rizzo MI, De Virgilio A (2015). Goodpasture's syndrome: a clinical update. Autoimmun Rev.

[23] Segelmark M, Hellmark T, Wieslander J (2003). The prognostic significance in Goodpasture's disease of specificity, titre and affinity of anti-glomerular-basement-membrane antibodies. Nephron Clin Pract.

[24] Kalluri R, Meyers K, Mogyorosi A, Madaio MP, Neilson EG (1997). Goodpasture syndrome involving overlap with Wegener's granulomatosis and anti-glomerular basement membrane disease. J Am Soc Nephrol.

[25] Siddiqi HK, Mehra MR (2020). COVID-19 illness in native and immunosuppressed states: a clinical-therapeutic staging proposal. J Heart Lung Transplant.

[26] Kronbichler A, Lee KH, Denicolò S (2020). Immunopathogenesis of ANCA-associated vasculitis. Int J Mol Sci.

[27] Liu Y, Sawalha AH, Lu Q (2021). COVID-19 and autoimmune diseases. Curr Opin Rheumatol.

